# Kinematic and muscle co-activation patterns in the dominant arm across forehand stroke phases in wheelchair tennis

**DOI:** 10.3389/fbioe.2024.1518091

**Published:** 2025-01-14

**Authors:** Khaled Abuwarda, Abdel-Rahman Akl

**Affiliations:** ^1^ Department of Physical Education and Kinesiology, College of Education, Qassim University, Qassim, Saudi Arabia; ^2^ Faculty of Physical Education-Abo Qir, Alexandria University, Alexandria, Egypt

**Keywords:** biomechanics, disability, EMG, wearable sensors, physical activity

## Abstract

**Objective:**

This study investigated upper limb kinematics and muscle co-activation in wheelchair tennis players during the forehand stroke. By analyzing linear and angular kinematic variables alongside muscle co-activation patterns, the study aimed to provide insights into the biomechanical mechanisms supporting forehand stroke performance.

**Method:**

Fifteen professional male wheelchair tennis players (height: 163.9 ± 2.05 cm; mass: 64.1 ± 3.07 kg; age: 32.2 ± 7.97 years) participated in this study. Electromyographic data from six muscles around the dominant arm joints were recorded using the Myon system. Four fixed GoPro Hero 8 cameras (120 Hz) captured 3D video, and kinematic analyses were performed using the APAS system. The forehand stroke was analyzed across three phases: (1) backswing, (2) forwardswing, and (3) follow-through.

**Results:**

The results showed significant phase-specific changes in muscle co-activation for the shoulder (*p* < 0.001), elbow (*p* < 0.005), and wrist (*p* < 0.01). Muscle co-activation was highest during the backswing phase, decreased during the forwardswing, and increased again during the follow-through phase. This pattern reflects the need for joint stability and control, particularly when changing stroke direction and slowing the arm after impact.

**Conclusion:**

These findings provide novel insights into the kinematic and neuromuscular mechanisms underlying the forehand stroke in wheelchair tennis. The data provide hypotheses about potential training and rehabilitation strategies that should be tested by prospective studies. The results also highlight the unique demands of wheelchair tennis, contributing to inclusive, evidence-based approaches to enhancing performance and safety in disability sports.

## 1 Introduction

Wheelchair tennis is a prominent Paralympic sport that follows similar rules to traditional tennis, with the key exception that players are allowed two ball bounces (Williamson et al., 2024). Despite the significant growth in Paralympic sports, there is limited research addressing the biomechanics and muscle activity in wheelchair tennis, particularly concerning the forehand stroke (Rietveld et al., 2024). The sport demands frequent directional changes and multidirectional mobility, which requires players to both propel their wheelchairs and handle a racket simultaneously. This combination of tasks introduces a layer of complexity, as players must perform high-intensity movements like sprinting, braking, and turning while maintaining racket control (Sanchez-Pay et al., 2023). Improving our understanding of the mobility patterns and stroke mechanics in wheelchair tennis is critical for optimizing player performance and creating tailored training programs (Rietveld et al., 2023).

The forehand stroke is especially important in tennis, as it allows players to generate high ball velocities and is typically used more frequently than the backhand in competitive play (Fernandez-Fernandez et al., 2010; Reid et al., 2016). The stroke consists of three phases: backswing, forwardswing, and follow-through, each involving specific biomechanical events (Genevois et al., 2020). Maintaining racket control and force transfer to the racket during these phases are essential to high-level performance (shot accuracy, ball speed and spin). However, repetitive stress placed on the upper limb during these movements also increases the risk of musculoskeletal injuries (Roetert et al., 2024). To address this, tennis players and coaches should not only work on enhancing overall forearm muscle strength but also prioritize balancing the strength of the wrist flexor and extensor muscles. This balance is particularly important for reducing impact loads associated with both Eastern and Western grips, ultimately improving performance, enhancing comfort, and minimizing the risk of sports injuries (Dong et al., 2024).

Wheelchair tennis athletes are particularly prone to overuse injuries in the shoulder due to the constant load applied during propulsion and stroke performance. This need to generate and transfer forces from the chair through the trunk, arm and racket can lead to muscle imbalances, especially in the scapular and shoulder complex, which increase the risk of shoulder injuries (Silva et al., 2010; Morrow et al., 2011). Furthermore, the elbow and wrist play crucial roles in transmitting force during the stroke. The elbow serves as a key joint in the kinetic chain of the upper extremity, transferring forces through the arm segments to the racket. Repeated stress on the elbow and wrist can impair force regulation and further contribute to injury risk (Heales et al., 2016; Ju et al., 2021).

Muscle co-activation, the simultaneous contraction of agonist and antagonist muscles, is crucial for joint stability, motor control, and injury prevention during high-intensity tasks like forehand strokes and gripping sport implements (Hortobagyi et al., 2009; Nagai et al., 2011). This is especially important for maintaining joint stiffness and controlling complex stroke movements (Garcia-Vicencio et al., 2024).

Recent advancements in motion capture technologies and wearable sensors have enabled more precise analysis of upper limb kinematics and muscle activity during sports performance (Shieh et al., 2016; Rum et al., 2021). These tools provide detailed insights into movement patterns, offering a deeper understanding of biomechanics, muscle activation patterns, and their implications for performance and injury risk. This knowledge is invaluable for optimizing training and rehabilitation strategies. However, despite these technological advancements, a gap remains in the literature regarding the interaction between muscle co-activation and kinematic variables during the various phases of the forehand stroke in wheelchair tennis.

Therefore, the purpose of this study is to investigate the kinematics and muscle co-activation patterns of the dominant arm during the forehand stroke in wheelchair tennis. We specifically sought to address the following research questions:

1. What are the key kinematic differences in the dominant arm during the different phases of a forehand stroke?
2. How do muscle activation levels vary among key muscles during the different phases of a forehand stroke?
3. What is the muscle co-activation observed between antagonistic muscle groups throughout the forehand stroke?

## 2 Materials and methods

### 2.1 Subjects and study design

In this study, we used G-Power software version 3.1.9.7 (Universität Kiel, Germany) to determine the necessary sample size. Assuming an effect size of 0.45, with a significance level (α) of 0.05 and a statistical power of 95%, the calculation indicated that 15 participants would be required. Fifteen professional male wheelchair tennis players (mean height: 163.9 ± 2.1 cm, mean mass: 64.1 ± 3.1 kg, mean age: 32.2 ± 8.0 years) participated in this study. All participants held official rankings in the Egyptian Tennis Federation and regularly competed in professional wheelchair tennis tournaments. The study adhered to the guidelines of the Declaration of Helsinki and was approved by the university’s institutional ethics committee. Before participation, all subjects provided written informed consent.

### 2.2 Experiment protocol

Following a 15-minute warm-up consisting of stretching exercises, protocol familiarization, and targeted shoulder and elbow mobility exercises. The participants were asked which limb they would prefer to use for the performance and exercises in order to identify which limb was dominant (Kotsifaki et al., 2021), and performed three successful forehand stroke attempts, each separated by 1-min rest intervals. The forehand stroke was analyzed across three phases: backswing, which begins at the initiation of the movement and ends with elbow extension; forwardswing, which starts with the arm’s forward motion and ends at ball release; and follow-through, which begins at ball release and continues until the completion of the movement. The study assessed three linear kinematic parameters (change ratio of resultant displacement, change ratio of resultant velocity, and change ratio of resultant acceleration) and three angular kinematic parameters at the sagittal plane (change ratio of angle, change ratio of angular velocity, and change ratio of angular acceleration) during each phase. Additionally, ball-related metrics such as ball height, ball velocity, and ball release angle after impact of the racket were measured. For each participant, the kinematic variables and co-activation index were averaged across three trials.

### 2.3 Kinematic recording and analysis

**FIGURE 1:**
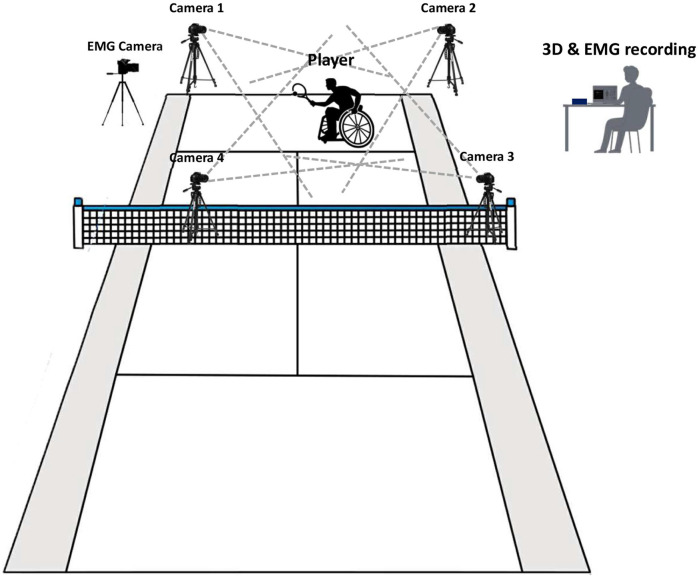
Schematic representation of the testing setup.

Seventeen reflective markers were applied to an-atomical landmarks using double-sided adhesive, including the anterior head, bilateral acromion processes, medial and lateral condyles of the humerus, styloid processes of the wrists, and the dorsum of the second and fifth metacarpal heads. Markers were also placed on the most lateral prominence of the greater trochanter. The 3D position of each marker was recorded at 120 Hz using four fixed GoPro cameras (GoPro HERO 8 Black; GoPro Inc., San Mateo, CA), synchronized using GoPro remote (see Figure 1).

**FIGURE 2:**
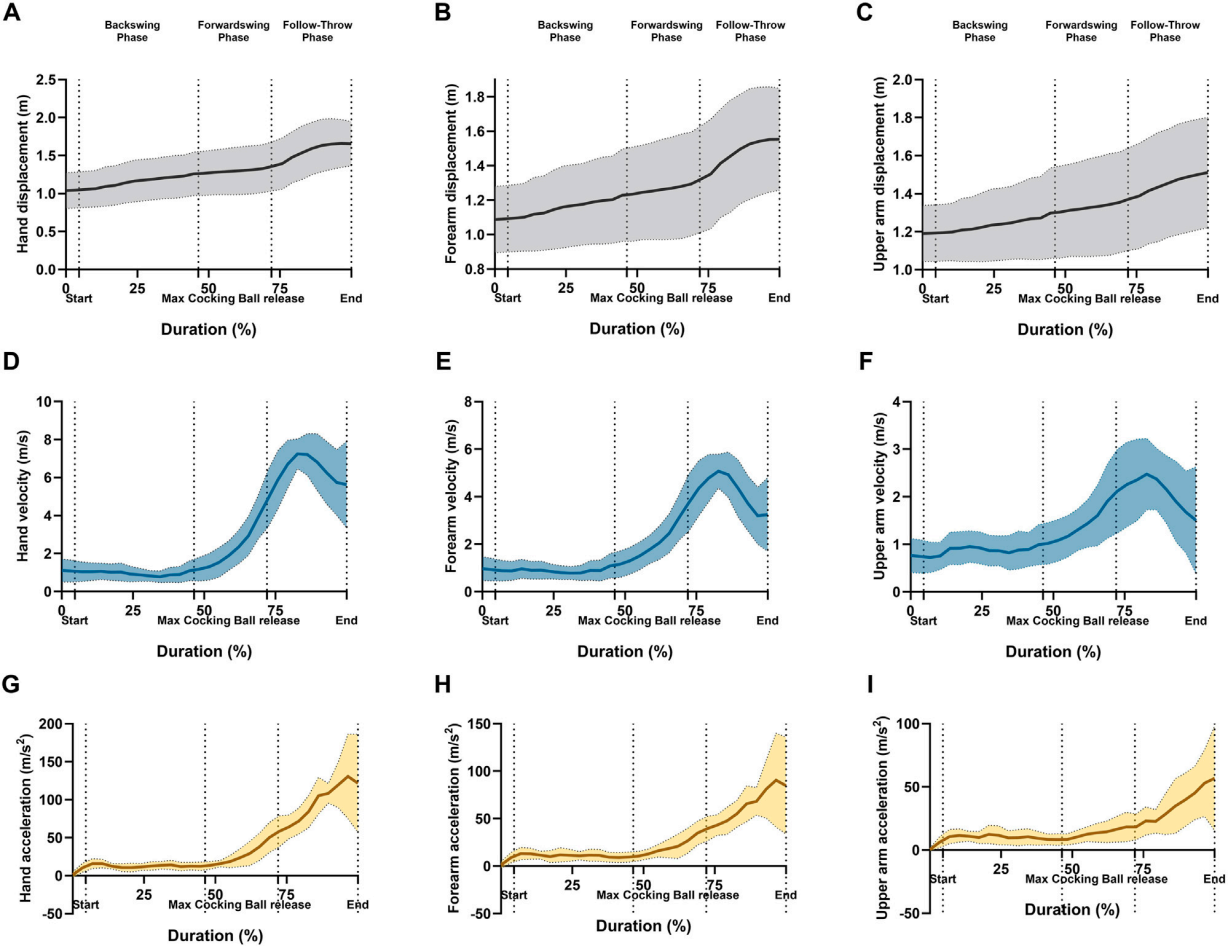
Graphical representations of the means and standard deviation for ankle angle of the linear kinematics; **(A)** hand resultant displacement, **(B)** forearm resultant displacement, **(C)** upper arm resultant displacement, **(D)** hand resultant velocity, **(E)** forearm resultant velocity, **(F)** upper arm resultant velocity, **(G)** hand resultant acceleration, **(H)** forearm resultant acceleration, and **(I)** upper arm resultant acceleration during forehand stroke phases (Backswing phase, Forwardswing phase, and Follow-Through phase).

**FIGURE 3:**
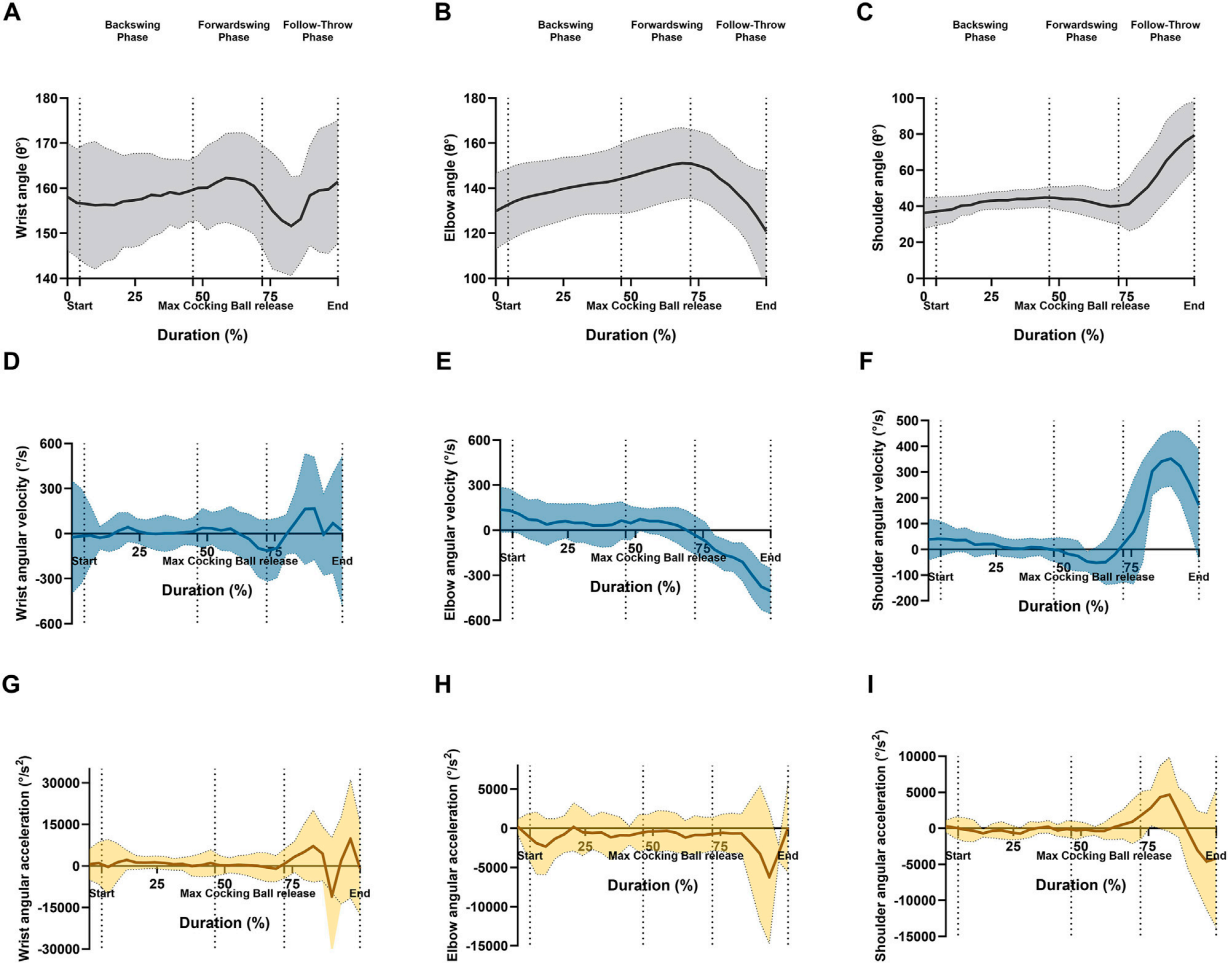
Graphical representations of the means and standard deviation for ankle angle of the angular kinematics at sagittal plane; **(A)** wrist angle, **(B)** elbow angle, **(C)** shoulder angle, **(D)** wrist angular velocity, **(E)** elbow angular velocity, **(F)** shoulder angular velocity, **(G)** wrist angular acceleration, **(H)** elbow angular acceleration, and **(I)** shoulder angular acceleration during forehand stroke phases (Backswing phase, Forwardswing phase, and Follow-Through phase).

The video recordings were digitized automatically using the APAS system (Ariel Dynamics, United States) and revised manually for data accuracy, then the kinematic data were filtered at 6 Hz using a 4th order low-pass Butterworth filter. Kinematic data was analyzed for three phases of the forehand stroke: (1) backswing, (2) forwardswing, and (3) follow-through. To compare variations, graphs depicting 3D kinematic outcomes (displacement, velocity, and acceleration) were generated (Figures 2, 3). The segment 3D coordinates were determined using the Direct Linear Transformation (DLT) according to the joint coordination system of the International Society of Biomechanics method (Abdel-Aziz and Karara, 2015) implemented in APAS. The local coordinate system for each segment was defined at the proximal joint center, the coordinate axes were defined: x = horizontal, y = vertical, and z = medial-lateral (Wu and Cavanagh, 1995; Soltani et al., 2016).

### 2.4 sEMG activity recording and analysis

In accordance with that surface electromyography (sEMG) was recorded using Myon m320RX sensors (Myon, Switzerland). The muscles monitored included the anterior deltoid (AD), posterior deltoid (PD), biceps brachii (BB), triceps brachii (TB), wrist flexor (WF), and wrist extensor (WE). The electrodes were positioned following SENIAM and Cram’s guidelines (Hermens et al., 2000; Criswell, 2010). Before electrode placement, the skin was shaved and cleaned. Bipolar surface electrodes (SKINTACT FS-RG1/10, Austria) were applied with an inter-electrode distance of 2 cm, in accordance with SENIAM guidelines (Hermens et al., 2000).

**FIGURE 4:**
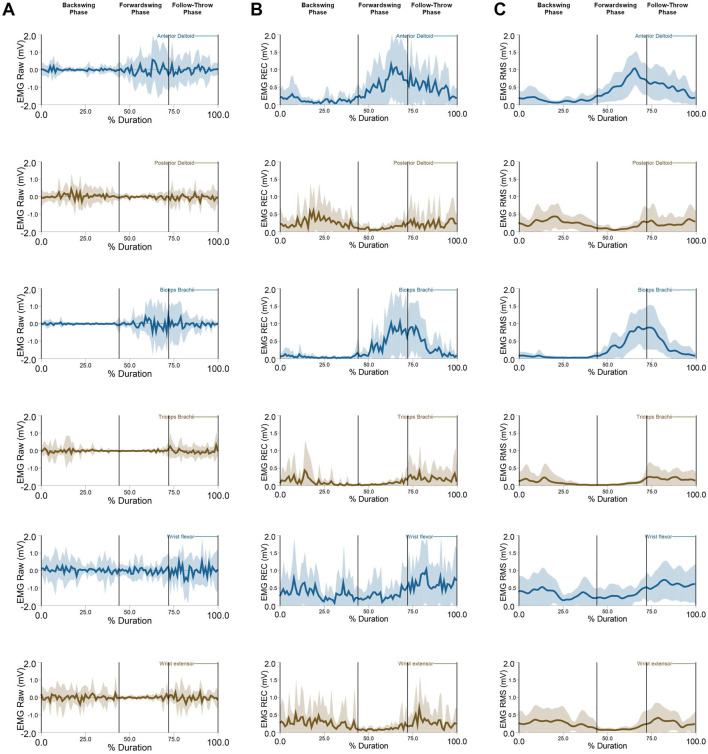
EMG data: **(A)** EMG raw, **(B)** EMG Rectified, **(C)** EMG RMS (M ± SD) of the anterior deltoid, posterior deltoid, biceps brachaii, triceps brachaii, wrist flexor, and wrist extensor during forehand stroke phases (Backswing phase, Forwardswing phase, and Follow-Through phase).

The EMG signals were sampled at a frequency of 1,000 Hz and converted into digital form using a 16-bit analog-to-digital (A/D) converter. An additional camera was used for recording a synchronized video with the EMG data using proEMG software (Myon 320, Schwarzenberg, Switzerland). This also ensured time synchronization between the data for determining the performance phases before next processing. Signal processing was performed using Visual3D (C-Motion Inc., MD, United States). To minimize motion artifacts, a high-pass Butterworth filter with a 25 Hz cutoff frequency was applied. The signals were then rectified and low-pass filtered at 15 Hz using a 100 ms window to obtain an enveloped EMG signal using the root mean square (RMS) (Figure 4) (Quittmann et al., 2020). EMG amplitudes were normalized to the maximum signal observed during the trials (NEMG) (Hermens et al., 2000).

### 2.5 Muscle co-activation index (CoI)

Muscle co-activation around the dominant arm joints was estimated using the Co-activation Index (CoI) Equation 1.

$$CoI=\frac{\int_{t_{1}}^{t_{2}}{NEMG}_{antagonist}\left(t\right)\;dt}{\int_{t_{1}}^{t_{2}}{\left[N\right.EMG}_{agonist}+N{EMG}_{antagonist}]\left(t\right)\;dt}\times 100$$ (1)

where t_1_ and t_2_ represent the beginning and end of each phase, NEMG_antagonist_ denotes the activity of the antagonist muscle, and NEMG_agonist_ denotes the activity of the agonist muscle during the backswing, forwardswing, and follow-through phases, separately (Kellis et al., 2003; Oliveira et al., 2017; Akl et al., 2021; Waanders et al., 2021).

### 2.6 Statistical analysis

Descriptive statistics, including means, standard deviations, and 95% confidence intervals were calculated. Data distribution was assessed using the Shapiro-Wilk test, confirming the suitability for parametric analysis. Differences between phases were analyzed using one-way repeated-measures analysis of variance (RM ANOVA). Post hoc comparisons were performed using Sidak tests. All statistical analyses were conducted using IBM SPSS Statistics v27 (IBM Corp., NY, United States).

## 3 Results

**FIGURE 5:**
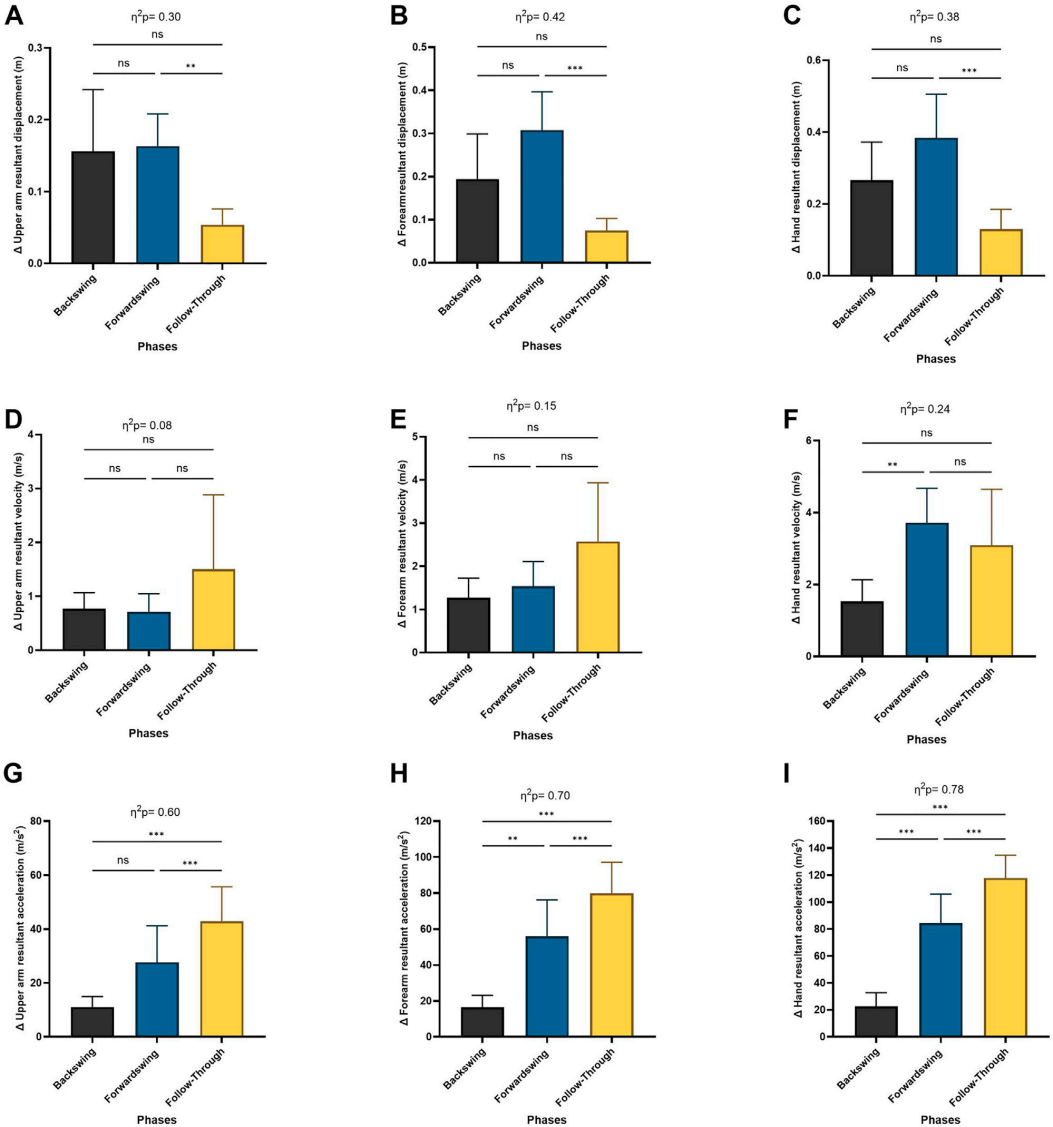
Pairwise comparisons associated with the significant main effects from the RM-ANOVA with mean values and coefficient interval for the change ratio (∆) of the linear kinematics during forehand phases; **(A)** upper arm resultant displacement, **(B)** forearm resultant displacement, **(C)** hand resultant displacement, **(D)** upper arm resultant velocity, **(E)** forearm resultant velocity, **(F)** hand resultant velocity, **(G)** upper arm resultant acceleration, **(H)** forearm resultant acceleration, and **(I)** hand resultant acceleration. Partial eta squared (η2p) and asterisk signs represent significant differences between phases: (***) indicates *p* < 0.001, (**) indicates *p* < 0.01, (*) indicates *p* < 0.05, and (ns) indicates non-significant.

The mean values and standard deviations for the ball height (0.87 ± 0.18 m), ball release velocity (16.09 ± 1.60 m/s), and ball release angle at sagittal plane (13.1° ± 4.3°) during the wheelchair tennis forehand stroke. For segment displacement in the dominant arm (upper arm, forearm, and hand), a significant main effect of phase was observed (*p* < 0.05, *p* = 0.001, *p* < 0.005, respectively; Figures 5A–C). Post hoc tests revealed significant decreases in displacement between the forwardswing and follow-through phases for the upper arm, forearm, and hand (*p* = 0.002, *p* < 0.001, *p* < 0.001, respectively). However, no significant variation was found between the backswing and forwardswing phases, or between the backswing and follow-through phases.

For hand velocity, the Repeated Measures ANOVA showed a significant main effect of phase (*p* < 0.05). Post hoc analysis revealed significant increases in hand velocity between the backswing and forwardswing phases (*p* = 0.01), with no significant differences between the forwardswing and follow-through phases, or between the backswing and follow-through phases. No significant variation was observed in upper arm or forearm velocities between the phases (Figures 5D–F).

Regarding segment acceleration in the upper arm, forearm, and hand, the Repeated Measures ANOVA indicated a significant main effect of phase (*p* < 0.001; Figures 5G–I). Post hoc tests showed significant increases in acceleration between the backswing and forwardswing phases, the forwardswing and follow-through phases, and the backswing and follow-through phases for the upper arm (*p* = 0.05, *p* < 0.001, *p* < 0.001), forearm (*p* = 0.006, *p* < 0.001, *p* < 0.001), and hand (*p* < 0.001), respectively.

**FIGURE 6:**
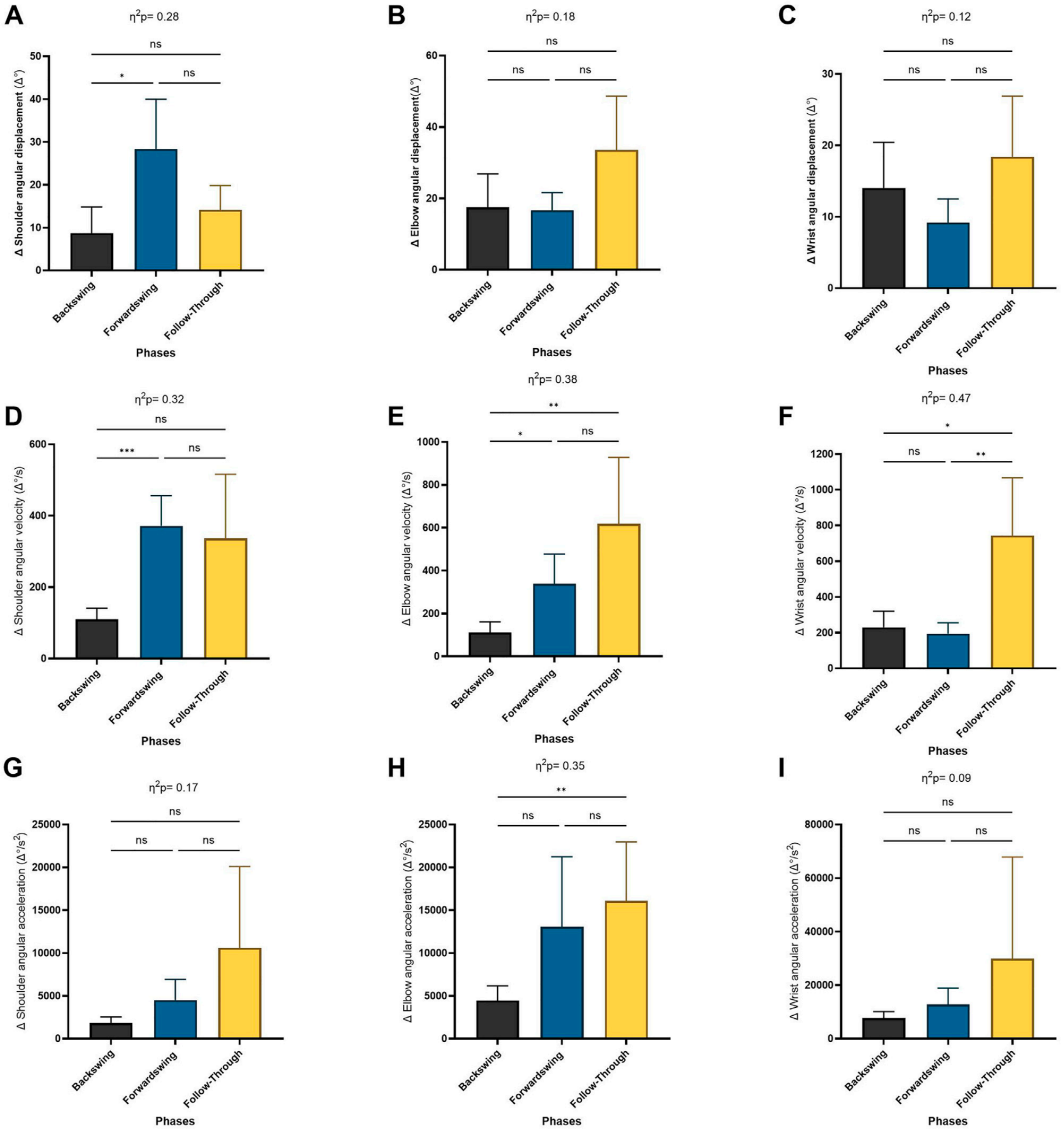
Pairwise comparisons associated with the significant main effects from the RM-ANOVA with mean values and coefficient interval for the change ratio (∆) of the angular kinematics during forehand phases; **(A)** shoulder angle, **(B)** elbow angle, **(C)** wrist angle, **(D)** shoulder angular velocity, **(E)** elbow angular velocity, **(F)** wrist angular velocity, **(G)** shoulder angular acceleration, **(H)** elbow angular acceleration, and **(I)** wrist angular acceleration. Partial eta squared (η2p) and asterisk signs represent significant differences between phases: (***) indicates *p* < 0.001, (**) indicates *p* < 0.01, (*) indicates *p* < 0.05, and (ns) indicates non-significant.

For the shoulder’s angular change ratio, a significant main effect of phase was found (*p* < 0.05), though no significant differences were observed in the elbow or wrist angular change ratio (Figures 6A–C). Post hoc analysis indicated a significant increase in the shoulder’s angular change ratio between the backswing and forwardswing phases (*p* = 0.04). However, no significant differences were seen between the forwardswing and follow-through phases, or between the backswing and follow-through phases for shoulder angular change ratio. Similarly, no significant variations were observed across phases for elbow and wrist angular change ratios.

The Repeated Measures ANOVA also revealed a significant main effect of phase for angular change ratio of velocity in the shoulder, elbow, and wrist joints (*p* = 0.01, *p* = 0.004, *p* = 0.002, respectively; Figures 6D–F). Post hoc tests showed significant increases in the shoulder and elbow angular change ratio of velocity between the backswing and forwardswing phases (*p* < 0.001, *p* = 0.03). Furthermore, significant increases in wrist angular velocity were observed between the forwardswing and follow-through phases (*p* = 0.005). Significant increases were also observed in the angular change ratio of velocity for the elbow and wrist between the backswing and follow-through phases (*p* = 0.008, *p* = 0.014). However, no significant variation was found between the backswing and forwardswing phases in wrist angular velocity, or between the backswing and follow-through phases for shoulder and wrist angular velocity. Similarly, no significant differences were observed between the forwardswing and follow-through phases for shoulder and elbow angular velocity.

For angular acceleration in the elbow joint, a significant main effect of phase was observed (*p* = 0.005; Figures 6G–I). Post hoc analysis indicated significant increases in elbow angular acceleration between the backswing and follow-through phases (*p* = 0.006). However, no significant variation was found between the backswing and forwardswing phases or between the forwardswing and follow-through phases for angular acceleration in any arm joints. Additionally, no significant differences were observed between the backswing and follow-through phases for shoulder and wrist angular acceleration.

**FIGURE 7:**
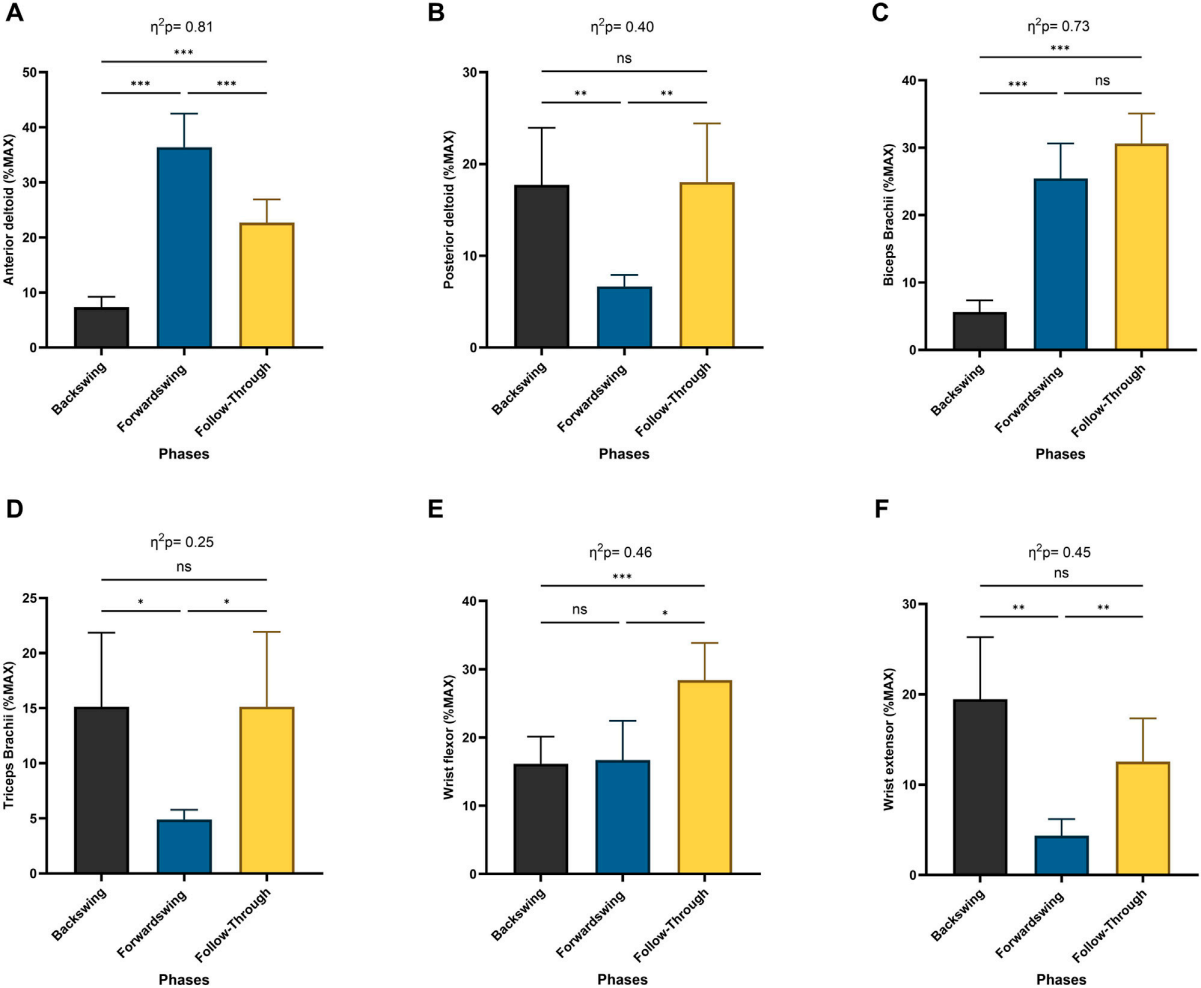
Pairwise comparisons associated with the significant main effects from the RM-ANOVA with mean values and coefficient interval for the normalized EMG (% MAX) of the dominant arm muscles; **(A)** anterior deltoid, **(B)** posterior deltoid, **(C)** biceps brachaii, **(D)** triceps brachaii, **(E)** wrist flexor, and **(F)** wrist extensor during forehand stroke phases (Backswing phase, Forwardswing phase, and Follow-Through phase). Significant differences for the *post hoc* tests between phases: (***) indicates *p* < 0.001, (**) indicates *p* < 0.01, (*) indicates *p* < 0.05, and (ns) indicates non-significant.

The Repeated Measures ANOVA revealed a significant main effect of phase on muscle activation across various muscles: the anterior deltoid (*p* < 0.001; Figure 7A), posterior deltoid (*p* < 0.01), biceps brachii (*p* < 0.001; Figure 7C), triceps brachii (*p* < 0.05; Figure 7D), wrist flexor (*p* < 0.001; Figure 7E), and wrist extensor (*p* < 0.01; Figure 7F). Post hoc analyses showed that anterior deltoid activation significantly increased between the backswing and forwardswing (*p* < 0.001) and backswing and follow-through phases (*p* < 0.001) but decreased between the forwardswing and follow-through phases (*p* < 0.001). For the posterior deltoid, activation decreased between the backswing and forwardswing phases (*p* < 0.01) and increased between forwardswing and follow-through phases (*p* < 0.01), with no significant change between backswing and follow-through. Biceps brachii activation significantly increased between the backswing and forwardswing (*p* < 0.001) and backswing and follow-through phases (*p* < 0.001), with no change observed between forwardswing and follow-through. Triceps brachii activation decreased significantly between backswing and forwardswing (*p* < 0.05) and increased between forwardswing and follow-through phases (*p* < 0.05), with no significant difference between backswing and follow-through. Wrist flexor activation increased significantly between backswing and follow-through (*p* < 0.001) and forwardswing and follow-through (*p* < 0.05), with no significant change between backswing and forwardswing. Similarly, wrist extensor activation decreased between backswing and forwardswing (*p* < 0.01) and increased between forwardswing and follow-through phases (*p* < 0.01), with no significant difference between backswing and follow-through. These findings highlight phase-specific variations in muscle activation during movement.

**FIGURE 8:**
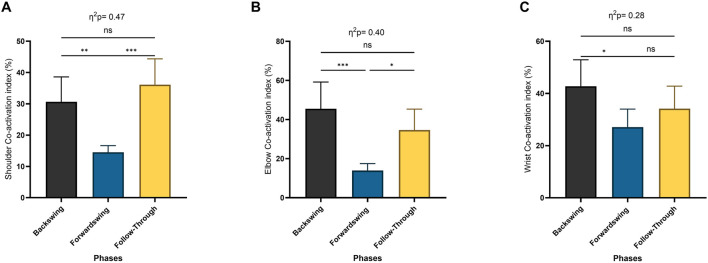
Pairwise comparisons associated with the significant main effects from the RM-ANOVA with mean values and coefficient interval for the co-activation index (CoI) (%) per phase of muscles around dominant arm joints; **(A)** shoulder, **(B)** elbow, and **(C)** wrist. Partial eta squared (η2p) and asterisk signs represent significant differences between phases: (***) indicates *p* < 0.001, (**) indicates *p* < 0.01, (*) indicates *p* < 0.05, and (ns) indicates non-significant.

The Repeated Measures ANOVA revealed a significant main effect of phase for shoulder co-activation (*p* < 0.001; Figure 8A), elbow co-activation (*p* < 0.005), and wrist co-activation (*p* < 0.01; Figure 8C). Post hoc analysis indicated a significant decrease in shoulder co-activation between the backswing and forwardswing phases (*p* < 0.01) and a significant increase between the forwardswing and follow-through phases (*p* < 0.001). No significant difference was observed between the backswing and follow-through phases. Elbow co-activation also showed significant decreases between the backswing and forwardswing phases (*p* < 0.001) and significant increases between the forwardswing and follow-through phases (*p* = 0.01). Additionally, wrist co-activation demonstrated significant decreases between the backswing and forwardswing phases (*p* < 0.05), but no significant changes were found between the other phases.

## 4 Discussion

### 4.1 Kinematics analysis and stroke mechanics

This study provides a detailed exploration of the biomechanical demands of the wheelchair tennis forehand stroke, with a focus on muscle co-activation and kinematics. The findings revealed lower ball velocities, release angles, and heights, reflecting reduced mechanical loading on the dominant arm. This can be attributed to the absence of lower-body contribution, necessitating a greater reliance on upper-body musculature. Such reliance, particularly on internal rotators, may contribute to muscle imbalances and an increased risk of overuse injuries (Bernard et al., 2004; Moreno-Perez et al., 2018). These results underscore the need for training programs tailored to the unique biomechanical and physical demands of wheelchair tennis (Roetert et al., 2024).

The kinematic analysis revealed significant increases in hand velocity between the backswing and forwardswing phases, consistent with efficient kinetic energy transfer along the upper limb segments. Consistent with previous studies, these findings highlight the importance of angular velocity and acceleration during the forwardswing, particularly in the elbow, to generate high-quality forehand strokes (Ju et al., 2024). While upper-arm and forearm velocities remained relatively stable, these segments serve critical roles as conduits in the kinetic chain. Such segmental coordination is essential for generating high racket speeds by transferring the force from the upper arm to the forearm, ultimately contributing to ball velocity (Ben Kibler, 1995).

Angular acceleration and change ratios varied significantly across phases, particularly in the shoulder and elbow. During the forwardswing, increased angular velocity and acceleration in these joints play pivotal roles in driving force through the kinetic chain. High acceleration rates during the follow-through phase reflect the critical need for energy dissipation and joint control. This is particularly important for wheelchair athletes who face additional physical demands, aligning with prior research on tennis stroke mechanics (Goosey-Tolfrey and Moss, 2005). The wrist’s increased angular velocity during the follow-through highlights its importance in stroke precision and stabilization. This finding aligns with research emphasizing the wrist’s role in fine-tuning ball trajectory and spin in tennis (Martin, 2018). The combination of joint acceleration and angular velocity emphasizes the wrist’s contribution to the overall kinetic chain.

### 4.2 Muscle activation levels

The results highlight distinct phase-specific variations in muscle activation, emphasizing the biomechanical demands of each phase of movement and the coordinated interplay between muscles. The anterior deltoid showed low activation during the backswing phase and increased during forwardswing phase, then declining in the follow-through phase. This pattern suggests that the anterior deltoid plays a primary role in forward motion but becomes less engaged during the initiating and deceleration phase (Mayrhuber et al., 2022; Abuwarda and Akl, 2023). In contrast, the posterior deltoid exhibited a complementary pattern, with reduced activation during the forwardswing but increased engagement in the follow-through, likely contributing to deceleration and posterior stabilization (Ju et al., 2021).

The biceps brachii activation peaked during the transition from the backswing to the forwardswing, maintaining this engagement into the follow-through phase, indicating its role in flexion and dynamic control throughout the movement (Furuya et al., 2021). Meanwhile, the triceps brachii showed decreased activation during the forwardswing but a subsequent increase in the follow-through, underscoring its contribution to controlled extension and stabilization as the motion concludes (Rota et al., 2012; Abuwarda and Akl, 2023).

The wrist flexors and extensors exhibited complementary activation patterns. The flexors showed significant engagement during the performance phases, with increased activation during the follow-through phase to enhance racket control. Conversely, the extensors peaked during the backswing phase, decreased during the forwardswing phase, and increased again during the follow-through. This coordination highlights their combined role in fine motor control and wrist stabilization, ensuring precision and effective force transmission throughout the motion (Furuya et al., 2021; Rietveld et al., 2024). Overall, these findings highlight the importance of muscle co-activation around the joints, which is essential for smooth and efficient transitions between movement phases.

### 4.3 Muscle Co-Activation patterns

Dynamic changes in shoulder muscle co-activation were observed across the phases of the stroke. Shoulder co-activation was significantly reduced during the forwardswing phase, followed by a marked increase during the follow-through. This phase-dependent variation reflects the coordinated interplay between the anterior deltoid (AD) and posterior deltoid (PD), which balance force generation and joint stability. The reduction during the forwardswing aligns with the need for energy transfer over stabilization in high-velocity movements (Knudson and Elliott, 2004). Increased co-activation during the follow-through phase stabilizes the shoulder and reduces strain on passive structures (Ben Kibler and Sciascia, 2004). The dynamic interplay between these muscles is essential for joint control and the smooth execution of the stroke (Abuwarda and Akl, 2023).

The elbow joint exhibited distinct muscle activation patterns, with the biceps brachii (BB) transitioning from an antagonist during the backswing to an agonist in the forwardswing and follow-through. The triceps brachii (TB) maintained consistent activation throughout all phases, ensuring joint stability. High co-activation during the follow-through was particularly notable, as it aids in deceleration and protects the elbow from excessive stress, consistent with findings in previous study (Pincivero et al., 2019).

The wrist flexor (WF) exhibited high activation throughout the stroke, indicating its importance in maintaining racket control. During the forwardswing, the reduced co-activation of the WF and wrist extensor (WE) may reflect the wrist’s role in dynamic force transfer (Heales et al., 2016). Increased co-activation during the backswing and follow-through phases enhances stabilization, aiding in precise transitions and racket trajectory control. This finding supports the principle of kinetic energy transfer through the upper limb to optimize stroke precision.

## 5 Limitations

This study has several limitations that should be considered when interpreting the findings. First, the sample size is limited due to the small number of high-level wheelchair tennis players available. To gain more comprehensive insights and examine differences between male and female players, further recruitment of wheelchair tennis athletes of both genders is necessary. Additionally, the EMG data were normalized to the maximum value of the recorded signal, a common approach for analyzing dynamic muscle activations. However, this method complicates direct comparisons with studies that use normalization based on the percentage of maximum voluntary isometric contraction (%MVIC). Another limitation involves the distortion of linear and angular velocity and acceleration data caused by smoothing techniques during impact. Finally, the simulated forehand strokes may not fully capture the wide variability in stroke techniques used during actual match play.

## 6 Practical applications and future work

The observed muscle co-activation and kinematic patterns offer practical insights for improving training and injury prevention strategies in wheelchair tennis. Strength and conditioning programs should prioritize muscle balance and joint stability, particularly in the shoulder and elbow (Ben Kibler and Sciascia, 2004). For instance, strengthening the posterior deltoid, triceps brachii, and wrist extensors may help optimize stroke mechanics while minimizing injury risks. Furthermore, neuromuscular training to enhance wrist and elbow coordination during high-velocity phases can contribute to improved performance (Pincivero et al., 2019). Tailored rehabilitation programs may also focus on reducing muscle imbalances caused by the unique demands of wheelchair tennis. Comparative studies between wheelchair and able-bodied athletes could further refine these training recommendations. Future research should evaluate the long-term effects of biomechanical adaptations in wheelchair athletes and explore the role of technology-assisted interventions, such as wearable sensors, in tracking and enhancing performance.

## 7 Conclusion

This study provides crucial insights into the biomechanics and muscle activity of wheelchair tennis, specifically through the analysis of co-activation patterns and kinematics during the forehand stroke. The findings show significant differences in co-activation patterns across the stroke phases, with a notable increase in co-activation during the follow-through phase to enhance control and stability. Our results offer practical applications for enhancing performance, reducing injury risk, and developing tailored rehabilitation programs. Additionally, this research enhances our understanding of kinematics and muscular activity in wheelchair tennis by exploring the differences between performance phases. It supports safer practices and promotes more inclusive participation in sports, particularly for competitive wheelchair tennis players.

## Data Availability

The raw data supporting the conclusions of this article will be made available by the authors, without undue reservation.
